# The Treatment of Chronic Constipation

**Published:** 1892-10-08

**Authors:** 


### THE HOSPITAL CLINIC.

*[The Editor will be glad to receive offers of co-operation and contributions from members of the profession. All letters should be addressed to* The Editor, The Lodge, Porchester Square, London, W.]

### ST. BARTHOLOMEW'S HOSPITAL.

### The Treatment of Chronic Constipation.

Before proceeding to deal with the treatment of constipation, it will be necessary to say a few words as to its causes. In the first place, it is not intended in this article to speak at all of constipation due to any form of organic disease of the bowel, such as rectal stricture and the like. The other causes may be divided into two classes, viz., I. Those arising from inefficient peristaltic action. II. Those associated with diminished secretion or excessive absorption. Of the latter class we know but little, except that they exist, and it is from the point of view of inefficient peristalsis that most cases of chronic constipation have to be viewed. The causes of this are many. Foremost among them, at any rate in large towns, is probably sedentary habits, among women anæmia, especially chlorotic anæmia, is a very common cause. The habitual use of large quantities of very strong tea among the poorer people and frequent resort to aperients may also be mentioned.

The treatment of chronic constipation as carried out at St. Bartholomew's Hospital will be dealt with under three heads, viz.: I. General hygienic treatment. II. Dietetic treatment. III. Medicinal treatment.

I. It is very difficult at a large general hospital to get those treated, who are mostly out-patients, to attend to this part of the treatment, and this for the obvious reason that nearly all of those we treat have to earn their daily bread, and are consequently at work from early morning till late in the evening. Those employed as clerks, and who, as a rule, sit nearly all day, are advised, as far as possible, to take exercise, such as by walking to their place of business in the morning instead of riding. More violent exercise, however, is generally believed to be better than walking, provided the patient can stand it, as this increases the activity of the liver, causes increased secretion of bile, which acts as a natural purgative. It has been mentioned above that constipation is almost invariably present in chlorotic girls. At a hospital like Bartholomew's large numbers of these attend as out-patients; many of them have come from country places, or, at any rate, from opportunities of exercise and the enjoyment of considerable chances of breathing fresh air, to long hours at workshops and factories of different kinds, as offices in the City, where the work is sedentary and the ventilation usually bad. These are advised to take every opportunity of getting fresh air in the parks and open spaces of London, or to try and get a holiday in the country. In addition to the above, patients are advised, if they are able, to take a cold bath in the morning, followed by friction with a rough towel, and to keep reasonable hours as to sleep, &c. Another most important thing is impressed upon patients, and that is the necessity for having a particular time for the bowels to be open, and, as far as possible, adhering to it, so that periodicity, which plays an important part in other bodily functions, may here also aid. Shortly after breakfast, as a rule, is found to be the best time. It is generally believed that neglect to daily attend to the calls of nature is a fruitful source of chronic constipation.

II. The dietetic treatment is often one of its most important branches, and advice on this point is constantly given at St. Bartholomew's. When combined with hygienic measures it is often found to be sufficient to cure the trouble. There should always be included in every diet some articles of food that have some solid residue, and here comes in the importance of vegetable food, either in the form of salad or cooked vegetables; these leave a certain amount of residue, which is undigested, and partly in a mechanical manner stimulate the large intestine to peristaltic action. Oatmeal porridge and brown wholemeal bread is also often recommended for the same reason. Fruit is also very good, and one frequently hears such things as stewed apples, prunes, or figs ordered or advised; the latter seem to have a greater effect if given on an empty stomach in the morning before breakfast. It must always, too, be remembered that a sufficiency of fluid must be taken in the course of the day; some people habitually take too little. Another measure often advised is taking a glass of cold or warm water immediately on rising in the morning, or during dressing.

III. As to medicinal treatment, it must first be noted that it is generally thought that the habitual use of the more powerful purgatives only tends to aggravate the complaint, as it is always found necessary to increase the dose, while the result obtained diminishes. It may, however, be necessary at the commencement of a course of treatment to get the bowel thoroughly unloaded first, and this is often done at Bartholomew's, either by one or two compound Colocynth pills followed by a black draught, or by one of these alone. The most common subsequent treatment is then to give aloes and strychnine in the form of a pill. Two of these pills are given every night to start with, and the patient is told that it is not improbable that there will be no result for the first day or two. When the pills do begin to act he must be made to understand that he is never to allow them to have a purgative action, but must omit taking one or more, so as just to produce a regular evacuation daily. This treatment is often most successful. The formula in common use is as follows: ℞ Pill of Barbadoes Aloes, gr. iv. ss.; Ext. of Nux Vomica, gr. ¼; treacle, quantum sufficit. Very frequently either the Pill Aloes et Ferri or Aloes et Myrrhæ are used instead of the above; the latter is most often given to women. This is practically Dr. Spender's well-known treatment. A very favourite prescription with some, especially to give to the older women, is equal parts of the Haustus Quassiae c̄ Ferro and Haustus, Menthae Sulphurium c̄ Menthae Sulphatis of the hospital pharmacopœia, which is equivalent to this formula: ℞ Acidi Sulphur. Diluti, ♏ v.; Syrupi Papaveris, ♏ xv.; Magnesii Sulphatis, gr. xxx.; Liquoris Ferri Perchloridi, ♏ xv.; Infusi Quassiæ, ℥js; Aquæ Menthæ Piperitæ, ℥js. is given once, twice, or three times a day, according to the effect produced. In giving iron to constipated people, its effect, of course, must be carefully watched for fear it should aggravate the trouble; but in chlorotic people its tonic effect is almost if not absolutely necessary. It is thought by many that its effect is increased and better results are obtained by the addition of some arsenic in the form of three to six minims of the Liq. Arsenicalis, and that the arsenic also assists in overcoming the constipation.

In the wards, Cascar Sagrada is very commonly used, one drachm of the liquid extract being given in the morning before breakfast, but by some its results are not thought to be very satisfactory. Mineral waters are, of course, as a rule, not much used in hospitals; but we have occasionally seen them used at Bartholomew's, more especially either Carlsbad or Hunyadi Janos. The former, however, has been generally prescribed in the form of Carlsbad Salts, one drachm, to be dissolved in half a glass of warm water, and taken in the morning before breakfast, or Sodium Sulphate has been given in place of the natural salts. The cases in which these are given are generally elderly people, who have lived fairly well in their day.

A word or two in closing on the use of enemata. Their use is not much encouraged at Bartholomew's for chronic constipation, and only in cases of older people are they, as a rule, advised. They should be used cold, as far as possible, or at any rate with just the chill off; and never really warm, as in that case they have no tonic effect on the rectum such as cold water has. The great difficulty about them is generally thought to be that their habitual use, instead of overcoming and curing the trouble, makes them ultimately become a necessity.

